# N1 Staging in Non-Small Cell Lung Cancer: Current Situation, Limitations, and the Importance of Peripheral Nodal Assessment

**DOI:** 10.3390/cancers18111792

**Published:** 2026-05-31

**Authors:** Tsukasa Ishiwata

**Affiliations:** Division of Respirology, Toronto General Hospital, University Health Network, Toronto, ON M5G 2C4, Canada; tsukasa.ishiwata@uhn.ca

**Keywords:** non-small cell lung cancer, N1 staging, TNM classification, thin convex probe endobronchial ultrasound, occult N1 disease, sublobar resection, peripheral lymph nodes

## Abstract

Accurate regional lymph node staging is essential for guiding treatment and predicting outcomes in non-small cell lung cancer. While the 9th edition of the TNM classification introduced prognostic subdivisions for N2 disease, the N1 category remains a single, unified descriptor. However, N1 disease is highly heterogeneous. Evidence shows significant survival differences between single-station (N1a) and multi-station (N1b) involvement, as well as between peripheral (N1p) and hilar (N1h) metastases. Standard medical imaging evaluation and conventional bronchoscopy often fail to detect “occult N1 disease,” leading to postoperative stage migration and suboptimal treatment sequencing. This diagnostic gap affects critical clinical decisions, including the selection of patients for sublobar resection, the administration of neoadjuvant chemoimmunotherapy, and the precision of radiation target volumes. The main obstacle to refining N1 staging has been the limited ability of existing clinical staging modalities to access and accurately assess N1p nodes. However, recent technological advances, particularly in thin convex probe endobronchial ultrasound examination, have renewed interest in bronchoscopic evaluation of N1p and in improving preoperative clinical N1 staging. The purpose of this review is to summarize the biological and immunological basis for N1 subclassification and evaluate how emerging technologies can bridge the gap between clinical and pathological staging. Refining the N1 compartment is vital for a personalized staging system that reflects the true biological spectrum of lung cancer.

## Simple Summary

Accurate regional lymph node staging is fundamental for the management of non-small cell lung cancer. While the 9th edition of the TNM classification refined N2 staging, the N1 category remains a single, undifferentiated entity due to the limitations of current clinical assessment methods. This review examines the biological and prognostic heterogeneity of N1 disease, specifically focusing on the distinction between hilar and peripheral nodal involvement. We address the clinical challenge of “occult N1 disease,” which often leads to unexpected postoperative stage migration and complicates the selection of candidates for sublobar resection or neoadjuvant chemoimmunotherapy. Furthermore, we evaluate the potential for advanced transbronchial nodal staging technologies to bridge the gap between clinical and pathological staging by accessing distal nodal stations. Refining N1 staging is essential for a personalized approach that optimizes the sequencing of systemic therapies and the precision of radiation planning.

## 1. Introduction

Lung cancer is the leading cause of cancer-related death worldwide [1]. Accurate staging is essential to guide treatment, predict patient prognosis, and standardize clinical trials. The Tumor, Node, and Metastasis (TNM) classification system, managed by the American Joint Committee on Cancer and the Union for International Cancer Control and updated by the International Association for the Study of Lung Cancer (IASLC), is the standard tool for risk stratification. Within this system, regional lymph node (N) involvement is one of the most critical prognostic factors in the management of non-small cell lung cancer (NSCLC). The N category (defined by absence or presence of lymph node metastasis) is based on anatomical location, extending from the lung (N1) to the ipsilateral mediastinum (N2) and to the contralateral or supraclavicular regions (N3) [2].

In January 2025, the 9th edition of the TNM classification was universally implemented [3]. A major refinement in this edition was the subdivision of N2 disease into N2a (single-station metastasis) and N2b (multi-station metastasis). This update was supported by survival analyses demonstrating a clear prognostic difference between single-station and multi-station N2 disease in both clinical and pathological staging. Pathological staging (pN) was determined through microscopic assessment of the resected specimens to verify the extent of lymph node metastasis at the time of surgical resection. For example, patients with pN2a disease showed a 5-year survival rate of 42%, whereas those with pN2b disease showed a 5-year survival rate of 31%. This statistical evidence prompted the American Joint Committee on Cancer and the Union for International Cancer Control to integrate N2a and N2b into the new stage groupings.

While the 9th edition successfully introduced prognostic subdivisions for the N2 category, the subdivision of the N1 category was deferred due to the diagnostic and statistical limitations of current clinical data. The N1 category includes metastasis to the ipsilateral, hilar, interlobar, lobar, segmental and subsegmental lymph nodes. Traditionally, the presence of N1 disease upstages patients to stage II or IIIA, indicating the need for adjuvant therapy after surgical resection [4]. Although adjuvant therapy is well-established and has significantly improved survival outcomes for N1 disease, the N1 definition has remained unchanged in the TNM classification since 1987. During the development of the 8th and 9th TNM editions [3,5], exploratory analyses suggested that quantifying N1 disease—specifically dividing it into single-station (N1a) and multi-station (N1b)—could provide valuable prognostic information. However, these proposals were rejected. The primary reason is a persistent gap between pathological precision and clinical capability: standard clinical staging tools, such as computed tomography (CT), positron emission tomography (PET), and conventional convex-probe endobronchial ultrasound (CP-EBUS), cannot reliably detect N1 subdivisions before surgery. Consequently, clinical N1 staging remains a “black box” characterized by high anatomical and prognostic heterogeneity. For example, a patient with a single metastasis in a distal subsegmental node (station 14) receives the same N1 classification as a patient with bulky hilar node involvement (station 10), even though their survival outcomes differ significantly. Furthermore, N1 lymph nodes are generally under-sampled and less studied compared to N2 nodes. Standard CP-EBUS cannot reach the more distal segmental and subsegmental nodes, resulting in less precise preoperative nodal staging [6].

Accurate, high-resolution N1 staging is now more critical than ever; the increasing use of lung-sparing sublobar resections for early-stage disease [7] and stereotactic body radiation therapy (SBRT) and the integration of neoadjuvant chemoimmunotherapy require precise initial staging. Relying on current medical imaging procedures and conventional bronchoscopy often results in missing occult N1 disease [8], which leads to unavoidable stage migration after surgery and carries a high risk of suboptimal treatment sequencing or undertreatment.

This review assesses the current limitations of N1 staging and the biological evidence supporting N1 sub-classification. Furthermore, we explore how emerging technologies, such as thin EBUS (TCP-EBUS), may finally bridge the gap between clinical and pathological staging. We also identify current knowledge gaps and offer recommendations for future trials and clinical practice.

### Literature Search

A comprehensive literature search was conducted in the PubMed and Embase databases for English-language peer-reviewed articles published up to March 2026. Search queries utilized combinations of MeSH terms and keywords, including: ‘non-small cell lung cancer’, ‘N1 staging’, ‘peripheral lymph nodes’, ‘TNM classification’, and ‘endobronchial ultrasound’. Articles were selected based on relevance to N1 anatomical or biological heterogeneity, prognostic differences between nodal substations, and recent bronchoscopic advancements, encompassing retrospective registry analyses, prospective validation trials, and international guidelines.

## 2. Anatomical Heterogeneity of N1 Lymph Nodes

**Figure 1:**
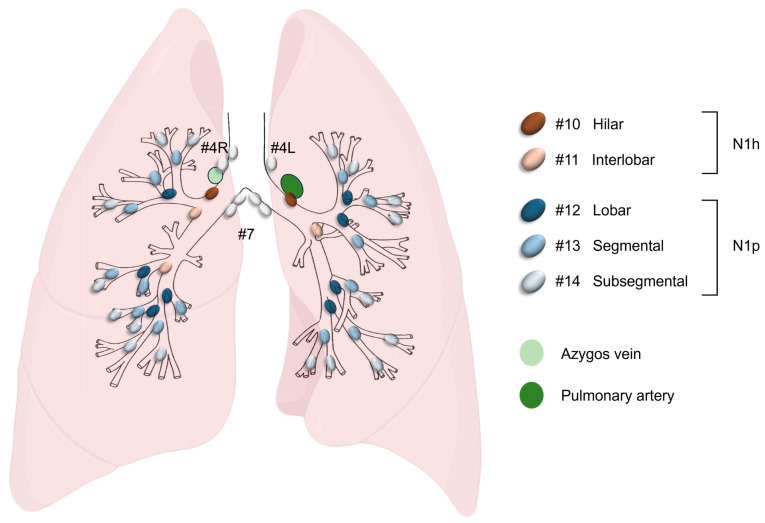
Anatomical distribution and classification of regional lymph nodes in non-small cell lung cancer. The N1 category is divided into the hilar/interlobar zone (stations 10 and 11, designated as N1h) and the peripheral zone (lobar, segmental, and subsegmental nodes, stations 12–14, designated as N1p).

To fully understand the N1 compartment, it is necessary to examine its anatomical and biological heterogeneity. The standardized IASLC lymph node map considers thoracic lymph nodes as 14 distinct stations to ensure a universal nomenclature [9]. The N1 category is broad, encompassing both the central zone (hilar and interlobar, stations 10 and 11, N1h) and the peripheral zone (lobar, segmental, and subsegmental, stations 12, 13, and 14, N1p) (Figure 1). Pulmonary lymphatic drainage typically follows a structured, centripetal pathway. Lymphatic fluid originates in the deep lung parenchyma and flows first through the peripheral nodes located at the bifurcations of the subsegmental and segmental bronchi. It then drains centrally into the interlobar and hilar nodes, and crosses the visceral pleura to enter the N2 mediastinal compartment. Due to this generally predictable drainage pattern, a peripheral lung tumor typically metastasizes to the segmental and lobar nodes before reaching the hilum. Consequently, patients with metastasis limited to the peripheral zone represent an earlier stage of disease and generally demonstrate superior survival outcomes compared to those with central hilar involvement [10]. Furthermore, pathological analyses of resected lung specimens show that the total number of peripheral lymph nodes is significantly higher in tumor-bearing segments compared to non-tumor-bearing segments [11]. This increase in lymph node or reactive lymphocytic node count is also observed when metastasis is present [12]. This localized lymphadenopathy suggests a strong, anatomically restricted immune response to the primary tumor [11]. Therefore, simply classifying a tumor as “N1-positive” is clinically inadequate. It fails to distinguish whether the metastasis is confined to the local subsegmental tissue or whether it has spread through the lobar lymphatic chain to the central hilum.

Limitations in probing peripheral lymphatics with current diagnostic tools often result in the identification of “occult” nodal disease upon surgical resection. Large-scale registry data and prospective surgical cohorts reveal that the incidence of occult metastases in patients staged as cN0 varies significantly depending on tumor size, surgical approach, and the extent of pathologic nodal evaluation [13,14,15]. For instance, in the CALGB 140503 randomized trial evaluating highly selected peripheral cT1a (≤2 cm) NSCLC, the overall intraoperative nodal upstaging rate was 6.4% (including both occult N1 and N2 disease) [14]. Conversely, the Society of Thoracic Surgeons database analysis of clinical stage I (cT1–T2a) tumors reported higher total upstaging rates of 11.6% for video-assisted thoracic surgery and 14.3% for open thoracotomy, with occult N1 disease specifically accounting for 6.7% and 9.3%, respectively [13]. Furthermore, national registry data from the National Cancer Database demonstrated that the detection of occult disease is strongly influenced by the extent of pathological nodal evaluation; the upstaging rate was 10.9% when 1 to 14 lymph nodes were pathologically assessed, but rose to 17.9% when more than 14 nodes were examined [15]. While all these historical multi-institutional databases shared a pragmatic definition of cN0 based on negative non-invasive imaging (CT and/or PET-CT), the wide variance from approximately 6% to 18% reflects differences in tumor size criteria, surgical implementation, and the rigorousness of formal lymphadenectomy rather than a single unified cohort phenotype.

## 3. The Microenvironment and Regional Immunological Interaction

The biological behavior of N1 metastasis shows significant histological variation. In patients with lung adenocarcinoma, the maximum diameter of the metastatic focus within the N1 lymph node is an independent prognostic factor. Studies have identified a critical threshold of 4 mm: patients with a metastatic focus of 4 mm or less in the N1 lymph node demonstrate a 5-year overall survival rate of 85.6%, whereas those with a focus larger than 4 mm have a survival rate of only 57.7% [16]. Notably, this size-dependent survival difference is not observed in squamous cell carcinomas, highlighting how different histological types exhibit distinct tumor microenvironments and biological behaviors.

The immunological environment of tumor-draining lymph nodes provides important insights into disease progression. These nodes are the primary anatomical sites where antigen-presenting cells present tumor antigens to naïve T cells, initiating the cancer-immunity cycle. Immunohistochemical profiling demonstrates that the immune microenvironment of hilar lymph nodes is substantially different from that of deeper peripheral nodes. Specifically, a relative depletion of effector CD4+ T cells within these regional nodes correlates directly with higher tumor Programmed Death-Ligand 1 expression [17]. This evidence suggests that the N1 compartment represents a major site of regional immunological interaction. When metastatic cells overcome the initial immune response in the peripheral nodes and advance to the central hilar nodes, it indicates both anatomical progression and the failure of the localized anti-tumor immune response. Accurately identifying N1p involvement is, therefore, more than just anatomical mapping [10]; it represents the detection of a specific biological and immunological state. Recognizing this state is essential for guiding aggressive, tailored therapeutic interventions before systemic immune tolerance develops.

## 4. Diagnostic Uncertainty and Modality Limitations

Lymph node involvement is a critical determinant of lung cancer management and survival. While the detection of N2 (mediastinal) metastases has improved, N1 (hilar and intrapulmonary) nodes continue to present a diagnostic challenge. Standard non-invasive clinical staging relies heavily on contrast-enhanced CT and FDG-PET/CT [18]. However, both modalities have significant limitations in both spatial and metabolic resolution. CT scan evaluation primarily relies on a size threshold—typically a short-axis diameter greater than 10 mm—to identify malignancy [18]. This morphological criterion frequently misses aggressive micrometastases (pathologically defined as tumor deposits measuring between 0.2 mm and 2.0 mm) [19] in normal-sized nodes and over-stages hyperplastic nodes resulting from benign pulmonary infections. FDG-PET/CT improves diagnostic accuracy [20]. However, it frequently yields false-positive results due to localized inflammation or granulomatous diseases (e.g., sarcoidosis, tuberculosis). Furthermore, false-negative results are common in cases of indolent tumors (e.g., mucinous adenocarcinomas, carcinoid tumors) or nodes with low metabolic activity [21]. For nodes with a standardized uptake value of less than 4, the sensitivity for detecting malignancy drops significantly [22]. In a prospective study of 400 lung cancer patients, FDG-PET detected only 71% of N1 metastases, compared to only 43% by CT [23]. Given the high prevalence of occult N1 disease, European guidelines strongly recommend invasive staging, such as EBUS-guided sampling or mediastinoscopy, for any patient with suspected N1 involvement [24].

## 5. Pathologic Nodal Staging and Intraoperative Assessment

Pathologic nodal staging in lung cancer depends heavily on thorough nodal retrieval, processing, and sectioning, with its completeness determined by both the specimen type and the surgical procedure. In small biopsy specimens, such as those from EBUS-guided sampling, staging accuracy is inherently limited by the focal nature of tissue acquisition; consequently, small metastatic foci may be missed despite technically adequate sampling. In contrast, for surgical resection specimens, nodal upstaging can be optimized through meticulous gross examination, complete embedding of submitted nodes, and serial sectioning, as standard single-plane evaluation frequently misses micrometastases [6,19].

Major oncology and surgical guidelines—including the American College of Surgeons Commission on Cancer, the National Comprehensive Cancer Network, the European Society of Thoracic Surgeons (ESTS), and IASLC—converge on the principle that accurate lymph node staging in curative-intent lung cancer surgery requires a deliberate, multi-station, and anatomically informed assessment rather than a simple node count. All organizations agree that incomplete evaluation leads to occult understaging and inferior survival. However, there is no global consensus regarding the specific N1 stations or precise number of nodes required for definitive dissection or sampling. For N1 nodal evaluation, the Commission on Cancer [25] and the National Comprehensive Cancer Network [26] mandate the assessment of at least one N1 station and emphasize intraoperative assessment to overcome the limitations of preoperative imaging. Conversely, while the ESTS guidelines do not mandate a specific count for an N1 lymph node to be retrieved during systematic nodal dissection, they strongly recommend the routine dissection of both hilar and intrapulmonary lymph nodes to ensure oncological completeness [19].

The scope of intraoperative lymph node assessment also varies significantly by the type of surgical procedure. For lobectomy, guidelines and staging literature strongly support systematic hilar and mediastinal evaluation as an essential component of accurate pathologic staging [19]. However, with the increasing adoption of segmentectomy for selected early-stage tumors, nodal staging is often less complete [27]. Occult N1 disease can easily go undetected if hilar and intrapulmonary nodes are not fully examined. Consequently, a pN0 designation following segmentectomy must be interpreted with caution, taking into critical account the exact extent of the N1 lymph node dissection and subsequent histologic examination actually performed.

## 6. Biological Heterogeneity of N1 Disease: Hilar (N1h) Versus Peripheral (N1p)

N1 metastasis significantly impacts prognosis. Patients with pN1 NSCLC have a 5-year survival rate of approximately 58%, compared to 83% for those with pN0 disease [3]. However, as described above, N1 is not a biologically homogeneous category. N1 nodes are anatomically divided into the hilar/interlobar zone (stations 10 and 11, designated as N1h) and the peripheral zone (stations 12, 13, and 14, designated as N1p) (Figure 1).

Decades of research have demonstrated a profound prognostic divergence between these zones. N1h tumoral involvement may be associated with an aggressive clinical behavior, whereas N1p involvement is typically more indolent. An early study showed that 5-year survival for station 10 involvement tended to be poorer than for N1p (station 12–14) involvement (39% vs. 53%, *p* = 0.02) [28]. Another study reported that metastasis confined to station 13 and station 14 yielded superior overall survival curves compared to those with station 12 involvement (69% vs. 46%) [29]. Meta-analyses of pathologically confirmed N1 (pN1M0) NSCLC report a pooled 5-year overall survival of approximately 56% for isolated N1p involvement, compared to only 40% for N1h involvement [10] (Table 1). Notably, the hazard ratio for death is 1.67 times higher for patients with N1h disease.

## 7. The Prognostic Dichotomy: Single N1 (N1a) Versus Multiple N1 (N1b) Disease

The primary rationale for subdividing the N1 category is that the extent of lymph node involvement directly impacts prognosis. The number of metastatic lymph nodes and involved stations reflects the degree of lymphatic dissemination, which correlates with the overall disease burden and patient survival [3,5]. Large retrospective studies have repeatedly demonstrated that the current N1 category encompasses patient populations with distinctly different outcomes [3,5,30]. When pathological N1 disease is subdivided into N1a (involvement of a single N1 nodal station) and N1b (involvement of multiple N1 nodal stations), a clear divergence in long-term survival emerges.

A large-scale analysis of the Surveillance, Epidemiology, and End Results registry database, including 3234 patients with N1 and N2 NSCLC, showed that the 5-year cancer-specific survival rate was 49.7% for N1a disease [30]. In contrast, the survival rate was significantly lower, at 41.4%, for N1b disease (*p* = 0.00022), indicating that the number of involved nodal stations is a reliable marker of tumor behavior. Similar findings have been reported in multiple institutional cohorts [31,32,33]. Importantly, no significant difference in survival was observed between patients with N1b disease and those with single-station N2 disease without N1 involvement (N2a1). This observation has been consistently reported by multiple studies, suggesting that disease involving multiple N1 stations may represent a level of spread similar to “skip metastasis” to N2 nodes [31,32,33,34,35]. These findings highlight the biological and clinical importance of N1 subclassification.

However, the IASLC Committee elected not to adopt this N1 subclassification for the 9th edition of the TNM staging system. This decision was based on strict criteria established prior to the analysis; any modification to the TNM system must be supported by robust evidence that is consistent across different populations and valid in both pathological and clinical staging. While a clear difference was observed in the pathological dataset between pN1a and pN1b (*p* = 0.0088) (Table 2), no meaningful survival difference was seen between the cN1a and cN1b in the large clinical staging dataset (*p* = 0.8443) [3]. This lack of separation in clinical staging does not undermine the biological basis of N1 subclassification; rather, it reflects the limitations of current staging modalities [36]. With the tools available today, it remains challenging to accurately distinguish between single- and multi-station N1 involvement preoperatively.

It must be acknowledged that the clinical evidence supporting N1 subclassification is predominantly derived from retrospective, observational cohorts, which are inherently vulnerable to selection bias and significant confounding factors. Most notably, historical data are heavily confounded by intraoperative variations in surgical nodal dissection—specifically, the thoroughness of intrapulmonary lymphadenectomy across institutions—as well as unmeasured heterogeneity in adjuvant treatment paradigms and tumor histology. These limitations underscore why retrospective survival differences have not yet seamlessly translated into universally accepted clinical staging practice.

## 8. The TNM Staging Paradox and Technical Constraints

Despite the clear biological differences between hilar and peripheral N1 disease, as well as between single-station N1 and multi-station N1 involvement, the 9th edition of the TNM classification retained N1 as a single, unified descriptor [3]. This situation creates a clinical paradox: while it is established that N1 disease is heterogeneous, standard diagnostic practices necessitate treating it as homogeneous. As noted above, although the IASLC deferred subclassification of N1 due to a lack of significant differences in overall survival in clinically staged N1 disease (characterized by failure to reach a predefined threshold of statistical significance, *p* < 0.05), but not in pathologically staged N1 disease, it is worth considering that the limitations of current and historical staging methods may themselves be a contributing factor to this lack of statistical distinction.

Historically, clinical N1 staging has relied on radiological imaging. However, standard modalities often struggle to resolve sub-centimeter N1p nodes, leading to systematic under-staging and stage migration within databases such as IASLC. To address these inaccuracies, international guidelines from the ESTS [24] and the American College of Chest Physicians [37] now mandate tumor tissue microscopy diagnosis for accurate lung cancer staging. Endobronchial ultrasound-guided transbronchial needle aspiration (EBUS-TBNA) has replaced surgical mediastinoscopy as the gold standard for mediastinal metastatic disease staging, providing equivalent or superior diagnostic accuracy with significantly lower morbidity and healthcare costs [38,39,40]. Despite these advantages, the anatomical reach of conventional CP-EBUS is limited by its design. Standard CP-EBUS devices (e.g., Olympus BF-UC180F, Fujifilm EB-530US, and Pentax EB-1970UK) have distal end diameters ranging from 6.7 to 7.4 mm. While these scopes effectively access the central airways to sample the paratracheal (stations 2, 4), subcarinal (station 7), hilar (station 10), and interlobar (station 11) nodes, their size prevents advancement into the more distal bronchi. Because conventional CP-EBUS cannot navigate narrow, acutely branching segmental and subsegmental airways, lymph node stations 12, 13, and 14 have traditionally remained bronchoscopically inaccessible during routine preoperative evaluation. A new generation of CP-EBUS bronchoscopes (e.g., Olympus BF-UC190F, Fujifilm EB-710US, and Pentax EB19-J10U) has been introduced. These models feature slightly smaller diameters and improved angulation. A study showed that a newer EBUS bronchoscope, with distal end diameters as small as 6.6 mm, can successfully reach and diagnose peripheral pulmonary lesions [41]. However, to our knowledge, clinical evidence specifically regarding the use of these new-generation bronchoscopes for N1p biopsy remains limited. Consequently, patients staged as clinically node-negative (cN0) based on negative PET-CT imaging and negative conventional EBUS sampling frequently harbor undetected metastases in the peripheral zone.

## 9. Technological Evolution: The Advent of Thin Convex Probe-EBUS

**Figure 2:**
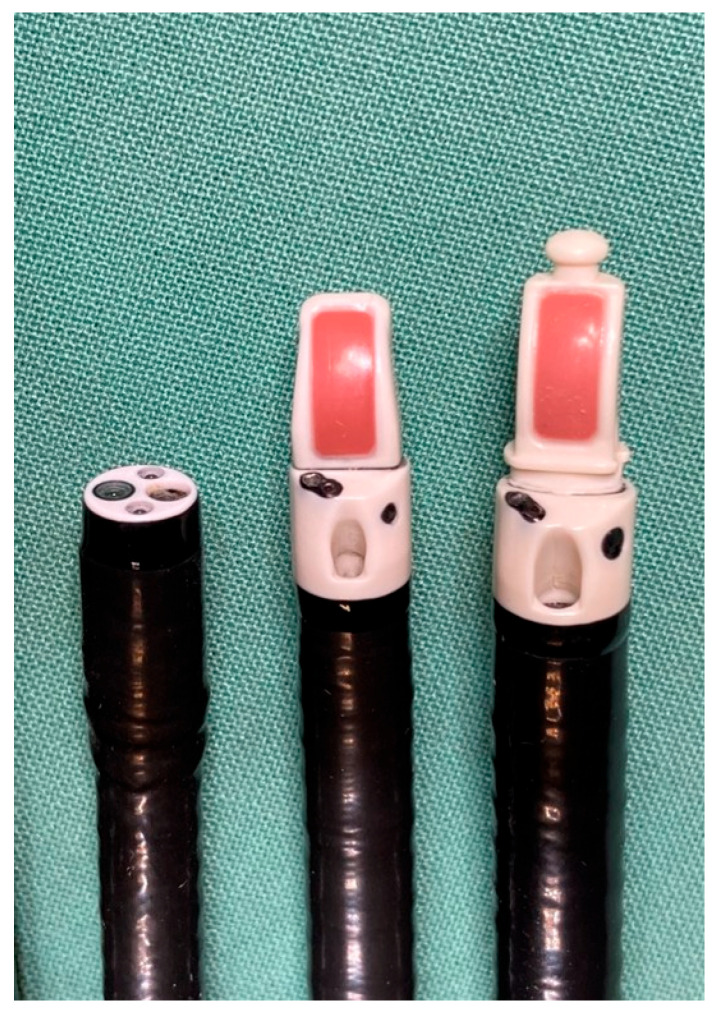
Thin convex-probe endobronchial ultrasound (TCP-EBUS) bronchoscope. From left to right: a standard bronchoscope (5.5 mm diameter, BF-H190, Olympus), a TCP-EBUS (5.9 mm diameter), and a conventional CP-EBUS (6.4 mm diameter).

**Figure 3:**
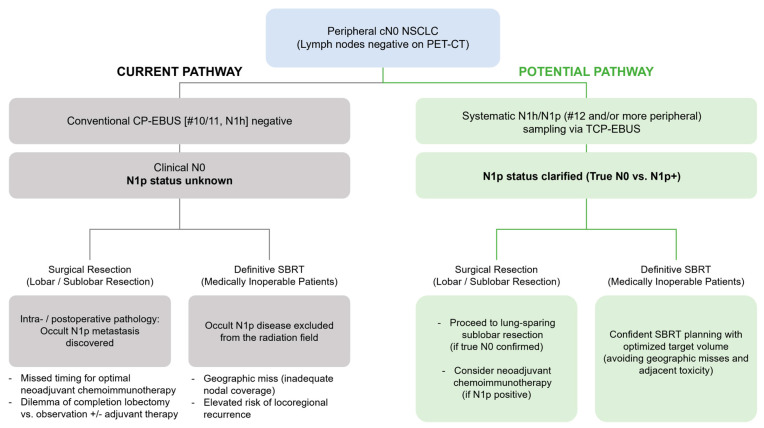
Current staging limitations of the N1 compartment and the potential clinical paradigm shift enabled by TCP-EBUS. The left pathway outlines the structural vulnerabilities of current standard practice, where the N1p status (Stations #12–14) remains preoperatively unknown. This diagnostic gap leads to unexpected postoperative upstaging in 6% to 18% of cases following intended sublobar resection, which consequently causes clinicians to miss the therapeutic window for optimal neoadjuvant chemoimmunotherapy. Alternatively, it results in geographic misses during definitive stereotactic body radiation therapy (SBRT). The right pathway illustrates a proposed preoperative workflow where systematic peripheral nodal sampling via thin convex probe endobronchial ultrasound (TCP-EBUS) achieves precise preoperative nodal stratification. This pre-therapeutic insight guides tailored management, allowing clinicians to confidently proceed with lung-sparing sublobar resection if true N0 is confirmed, consider neoadjuvant chemoimmunotherapy if N1p positivity is detected, or optimize SBRT target volumes to avoid adjacent toxicities and locoregional recurrence. Note: The clinical efficacy, safety, and diagnostic performance of TCP-EBUS for peripheral N1 lymph nodes await formal validation in large prospective randomized trials.

The recent development of the Thin CP-EBUS (TCP-EBUS) addresses these limitations. The TCP-EBUS (e.g., Olympus BF-UCP190F) features a further reduced outer diameter (5.9 mm), a shorter rigid distal tip, and an increased upward angulation range [42]. The working channel remains 2.2 mm, which allows for the use of standard EBUS needles. Figure 2 shows a prototype model of a TCP-EBUS bronchoscope next to a new generation CP-EBUS and a standard bronchoscope. These design changes—a smaller tip, and greater bending capability—enable navigation into more distal airways. Preclinical models demonstrate clear advantages in distal access. A study using *ex vivo* human lungs demonstrated that, although results varied by segment (with deeper peripheral reach in the lower lobe bronchi), the TCP-EBUS bronchoscope showed bronchial reachability comparable to that of a 4.8-mm-diameter bronchoscope for at least three bronchial generations [42]. This suggests that the middle third of the lung field is likely accessible and that, theoretically, station 12 nodes or more distal nodes could be reached. In a first-in-human pilot study, fifty-one patients underwent bronchoscopy for non-central lung lesions. Among 44 lesions that standard CP-EBUS failed to reach, TCP-EBUS visualized 82% (36/44) and successfully obtained diagnostic samples in 61% (27/44) with no procedural adverse events reported [43]. If widely adopted, TCP-EBUS could transform clinical practice by enabling the bronchoscopic sampling of N1p nodes (Figure 3). By making it technically feasible to differentiate between single-station (N1a) and multi-station (N1b) disease, or to identify “skip metastases,” TCP-EBUS could refine preoperative staging. However, while a prototype TCP-EBUS successfully sampled subsegmental N1 nodes in an *ex vivo* model [44], clinical studies have not yet reported biopsy outcomes for N1p nodes using TCP-EBUS in live patients. Currently, there are no randomized trials demonstrating that TCP-EBUS improves staging outcomes or clinical workflows. Future research must compare clinical pathways with and without TCP-EBUS.

The introduction of TCP-EBUS would create new opportunities alongside significant clinical dilemmas. As previously noted [45], the ability to sample peripheral nodes does not automatically mandate their sampling in every case. The availability of this technology necessitates a re-evaluation of current staging algorithms, starting with the definition of “systematic nodal staging.” While current guidelines mandate systematic sampling of the mediastinum (N2/N3), extending this mandate to N1 stations 12–14 would significantly prolong procedure times and increase patient risk. Currently, there are no established criteria to identify N1p nodes as “suspicious” for metastasis; these nodes are often smaller than mediastinal nodes and poorly visualized on PET-CT due to adjacent cardiopulmonary background uptake. Future research must define specific sonographic criteria, such as B-mode features [46] or elastography [47], to guide targeted, selective sampling. Alternatively, automated image analysis using artificial intelligence could help identify highly suspicious metastatic lymph nodes [48]. It must be re-emphasized that the success rate, sampling sufficiency, and diagnostic accuracy of TCP-EBUS for N1p nodes remain to be validated in large prospective trials. Moreover, detecting target lymph nodes with EBUS in the peripheral airways is inherently more challenging, as no clear anatomical landmarks are available. Implementing TCP-EBUS for N1p diagnosis will thus require bronchoscopists to master new techniques.

From a cost-effectiveness perspective, TCP-EBUS offers the theoretical advantage of combining primary tumor diagnosis and comprehensive nodal staging into a single session. As described above, peripheral pulmonary nodules located in the middle lung zone are also accessible by TCP-EBUS [42,43]. This could reduce the need for multiple invasive procedures, such as separate navigational bronchoscopies or CT-guided transthoracic biopsies. While standard EBUS-TBNA is well-established as cost-effective compared to surgical mediastinoscopy [39,40], formal cost-effectiveness analyses for TCP-EBUS have not yet been published. The upfront costs of new equipment and training must be balanced against potential savings from preventing under-staging, avoiding unnecessary surgeries, and reducing cancer recurrences.

## 10. Clinical Flow and Therapeutic Impact: The Consequences of Undetected N1 Disease

Undetected N1p lymph node metastasis also impacts multidisciplinary thoracic oncology by influencing treatment planning, tumor board deliberations, and potentially patient survival. The consequences of inaccurate clinical staging are particularly evident in surgical decision-making, the timing of systemic therapies, and radiation planning.

### 10.1. Impact on Thoracic Surgery: The Sublobar Resection Controversy

The clinical significance of accurate N1p staging in thoracic surgery lies in preventing inappropriate selection for sublobar resection, which carries a high risk of local recurrence when occult nodal disease is missed. Trials such as JCOG0802/WJOG4607L [49] and CALGB 140503 [7] have established sublobar resection as a surgical option for carefully selected patients with peripheral, clinically node-negative tumors measuring 2 cm or less. The oncological safety of sublobar resection depends entirely on the absence of lymph node metastasis, while a segmentectomy removes the primary tumor along with immediate segmental (station 13) and subsegmental (station 14) nodes, leaving adjacent lobar and interlobar nodal basins intact [50]. Notably, in large trials such as CALGB 140503, intraoperative sampling of N1 and N2 lymph nodes (stations 4, 7, and 10 for right-side tumors and stations 5 or 6, 7, and 10 for left-sided tumors) was required. The absence of metastasis had to be confirmed via intraoperative frozen section analysis as a prerequisite for performing segmentectomy. However, intraoperative frozen section analysis is subject to sampling errors [51]. Approximately 6.2–12.8% [14,49] of carefully selected cN0 patients are ultimately found to have occult N1/N2 disease on final postoperative pathology. This creates a difficult clinical dilemma: whether the patient requires a completion lobectomy or whether segmentectomy combined with adjuvant therapy is sufficient. Some retrospective, propensity-matched studies suggest that segmentectomy with adjuvant therapy may offer overall survival comparable to lobectomy for unexpected pN1 disease [52,53]. Importantly, accurate preoperative N1 staging could help resolve diagnostic uncertainty, allowing surgeons to optimize the extent of resection preoperatively and avoid both intraoperative conversion and postoperative upstaging.

### 10.2. Impact on Systemic Therapy: Neoadjuvant Versus Adjuvant Approaches

In systemic therapy, the precise timing of immune checkpoint inhibitor administration hinges on accurate preoperative staging, as systemic anti-tumor immune responses are optimally primed prior to surgical lymphadenectomy. Prior to the advent of immunotherapy, the survival benefit of adjuvant chemotherapy over surgery alone was well established for resectable NSCLC. Although neoadjuvant chemotherapy also improved survival compared to surgical resection alone, there was no clear evidence demonstrating its superiority over adjuvant therapy in terms of overall or disease-free survival [54,55]. In that context, earlier detection of N1 disease merely shifted the timing of systemic therapy without fundamentally altering survival outcomes. However, the introduction of immune checkpoint inhibitors has transformed this treatment paradigm. Landmark clinical trials, including CheckMate 816 [56], KEYNOTE-671 [57], and NADIM II [58], have established neoadjuvant chemoimmunotherapy as a standard of care for resectable NSCLC. The efficacy of neoadjuvant immunotherapy relies heavily on the cancer-immunity cycle [59]. Intact tumor-draining lymph nodes are essential sites where dendritic cells activate tumor-specific CD8+ T cells [60]. Administering immunotherapy before surgical resection utilizes the full antigenic load of the intact tumor microenvironment to generate a systemic immune response [61]. If a patient is incorrectly staged as cN0 due to undetected N1p disease, they are routed directly to surgery. Once the tumor and regional lymph nodes are surgically removed, the primary sites of immune activation are lost [62], thereby bypassing the opportunity for neoadjuvant treatment. Therefore, accurate preoperative N1 staging is critical not just for logistical planning, but for providing the patient with the optimal biological opportunity to mount a systemic immunological response.

### 10.3. Impact on Radiation Oncology: Defining the Clinical Target Volume

In radiation oncology, the clinical value of definitive N1 staging lies in the precise delineation of the target volume, which balances maximum locoregional tumor control against the prevention of severe radiation-induced toxicities in healthy tissues. For patients who are medically inoperable or require definitive chemoradiotherapy, accurate N1 staging is essential for radiation planning. Modern techniques, like SBRT and intensity-modulated radiation therapy, deliver high doses of radiation to precise anatomical areas. The success of these therapies depends on the accurate delineation of the clinical target volume. If an occult N1 metastasis is excluded from the radiation field due to negative PET-CT imaging, it results in a “geographic miss” and subsequent locoregional recurrence [63]. Conversely, empirically expanding the radiation margins to cover potential occult disease increases the radiation dose to healthy lung, esophagus, and heart, which can lead to severe toxicities such as radiation pneumonitis [64]. The SEISMIC trial demonstrated that in 155 patients with locally advanced NSCLC—who were potential candidates for radical dose or high-dose palliative conventional radiotherapy, with or without chemotherapy—systematic endoscopic staging identified a lesser extent of mediastinal disease than PET in 39 participants (25%). This accurate staging resulted in the downstaging of 31 patients (20%) from cN2–3 to cN0–1, which enabled 20 patients to undergo curative-intent surgical resection; postoperative pathological staging confirmed pN0–1 status in every case [63]. Furthermore, *in silico* radiotherapy planning based on EBUS-defined volumes demonstrated significant median dose reductions to adjacent critical organs compared to traditional plans based on PET-identified disease extent alone. Specifically, dose reductions were observed for the esophagus (5.3 Gy, *p* = 0.0001), heart (0.5 Gy, *p* = 0.0001), spinal canal (3.3 Gy, *p* = 0.0006), and lungs (1.4 Gy, *p* = 0.0001). Extending systematic bronchoscopic nodal staging into the N1p stations would further refine target volumes, maximizing tumor control while minimizing toxicity to normal tissues.

## 11. Conclusions

Accurate regional lymph node staging is the primary determinant for estimating survival and sequencing therapy in the management of NSCLC. While the 9th edition of the TNM classification refined the prognostic categories for the N2 mediastinal compartment, the IASLC deferred the subdivision of the N1 compartment. This decision was not due to a lack of evidence regarding the biological and immunological differences between N1h and N1p metastases, but rather to the historical difficulty of evaluating the lung periphery preoperatively.

Currently, clinical N1 staging remains uncertain, and the high incidence of occult metastasis complicates surgical decisions for sublobar resections. Furthermore, staging inaccuracies may prevent eligible patients from receiving neoadjuvant chemoimmunotherapy. The introduction of TCP-EBUS addresses these diagnostic challenges by enabling interventional pulmonologists to evaluate N1p nodes. However, the detection rates, sampling efficacy, and diagnostic accuracy of TCP-EBUS for these peripheral nodes have not yet been established in a clinical setting.

Refining the staging of the N1p compartment is essential for developing a personalized staging system that reflects the biological spectrum of lung cancer. There is an increasing need to determine how to integrate this diagnostic information to improve patient survival.

## Figures and Tables

**Table 1:** Prognostic impact of N1 subclassification.

Pathological N1 Subclassification	Anatomical Stations	5-Year Overall Survival	Hazard Ratio (95% CI)	Prognostic Implication
N1p (Peripheral)	#12, 13, 14	56%	Reference	Favorable prognosis; biologically early stage of lymphatic spread
N1h (Hilar/Interlobar)	#10, 11	40%	1.67 (1.44–1.94)	Intermediate prognosis between pN1p and pN2 disease

CI, confidence interval; Data adapted from the meta-analysis by Hu et al. [10].

**Table 2:** Five-year overall survival according to pathological N-stage subclassification.

Pathological N-Stage	5-Year Overall Survival	Comparison	*p* Value
pN0	83%		
pN1 (all)	58%		
Single-station N1 (pN1a)	58%	pN1a vs. pN1b	0.0088
Multi-station N1 (pN1b)	52%		
pN2 (all)	47%		
Single-station N2 (pN2a)	51%	pN2a vs. pN2b	<0.0001
Multi-station N2 (pN2b)	40%		
pN3	28%		

Data adapted from Huang et al. [3] based on the IASLC database.

## Data Availability

All data analyzed during this study are included in this published article.

## Abbreviations

The following abbreviations are used in this manuscript:

CP-EBUS	Convex-probe endobronchial ultrasound
CT	Computed tomography
EBUS-TBNA	Endobronchial ultrasound-guided transbronchial needle aspiration
ESTS	European Society of Thoracic Surgeons
IASLC	The International Association for the Study of Lung Cancer
NSCLC	Non-Small Cell Lung Cancer
PET	Positron Emission Tomography
SBRT	Stereotactic body radiation therapy
TCP-EBUS	Thin convex-probe endobronchial ultrasound
TNM	Tumor, Node, and Metastasis
